# Efficient computation of stochastic cell-size transient dynamics

**DOI:** 10.1186/s12859-019-3213-7

**Published:** 2019-12-27

**Authors:** Cesar Augusto Nieto-Acuna, Cesar Augusto Vargas-Garcia, Abhyudai Singh, Juan Manuel Pedraza

**Affiliations:** 1 Physics department, Universidad de los Andes, Bogotá, South America Colombia; 2 Mathematics and Engineering department, Fundación universitaria Konrad Lorenz, Bogotá, South America Colombia; 3 Electrical and Compute Enginering Department, University of Delaware, Newark, Delaware USA

**Keywords:** Finite state projection, Stochastic hybrid systems

## Abstract

**Background:**

How small, fast-growing bacteria ensure tight cell-size distributions remains elusive. High-throughput measurement techniques have propelled efforts to build modeling tools that help to shed light on the relationships between cell size, growth and cycle progression. Most proposed models describe cell division as a discrete map between size at birth and size at division with stochastic fluctuations assumed. However, such models underestimate the role of cell size transient dynamics by excluding them.

**Results:**

We propose an efficient approach for estimation of cell size transient dynamics. Our technique approximates the transient size distribution and statistical moment dynamics of exponential growing cells following an adder strategy with arbitrary precision.

**Conclusions:**

We approximate, up to arbitrary precision, the distribution of division times and size across time for the adder strategy in rod-shaped bacteria cells. Our approach is able to compute statistical moments like mean size and its variance from such distributions efficiently, showing close match with numerical simulations. Additionally, we observed that these distributions have periodic properties. Our approach further might shed light on the mechanisms behind gene product homeostasis.

## Introduction

Stochastic modeling of bacterial cell division has been widely used in systems biology[1–4]. Basic problems concerning the stochastic nature of cell biology include modeling of cell size distributions[5], effects of fluctuations in division control in terms of population fitness[6] and auto-correlation and spectral analysis of division strategies through several generations[7]. The importance of a stochastic outlook of the cell division control has been highlighted in literature considering physiological implications that potentially affect DNA concentration, surface transport and biosynthesis rates, as well as proteome composition[8].

Stochastic models can achieve high level of detail. Nowadays, predictions of stochastic modeling have been challenged experimentally by increasingly accurate high-throughput measurements of cellular variables enabled by time-lapse imaging, image processing and micro-fluidic devices for fine environmental control. These experiments have elucidated division strategies in rod shaped microorganisms like bacteria[2, 3], yeast[9] and archea[10].

Stochastic models for bacterial division control aim to explain how bacteria decide when to split into two descendants. These models can be divided in two main groups: Discrete stochastic maps (DSM) and Continuous Rate Models (CRM)[11]. DSM, the most used, are based on the idea that at a phenomenological, coarse-grained level, a size regulation strategy can be studied using the properties of division events. Hence, the division strategy is a map that takes cell size at birth *s*_*b*_ to a targeted cell size at division *s*_*d*_ trough a deterministic function *s*_*d*_=*f*(*s*_*b*_) plus stochastic fluctuations that have to be assumed[1, 7].

Depending on the mapping *s*_*d*_=*f*(*s*_*b*_), or traditionally between the added size *Δ*=*s*_*d*_−*s*_*b*_ and *s*_*b*_, division strategies are classified into three main paradigms: one is the *timer* strategy, in which a cell waits for a fixed time, on average, and then divides (*Δ* decreases with *s*_*b*_). Another is the *sizer*, in which a cell grows until it reaches a certain volume[12]before dividing (*Δ* increases with *s*_*b*_). The third one is the *adder*, a recently observed division strategy [2, 13], in which the cell grows adding, on average, a fixed size since the last division event (*Δ* does not depend on *s*_*b*_).

In contrast to the simple description given by a DSM approach, continuous rate models (CRMs) explain not only these mapping but other interesting phenomena. CRM consider, besides discrete division events, the cell cycle dynamics. This class of models describes the division as a continous-time stochastic process with an associated division rate *h* (also known as splitting rate function) that sets the probability of division into an infinitesimal time interval. Currently, the main problem with CRM is that it is not obvious a priori how to parametrize the division rate *h* given experimental setups [11].

Here, we propose an efficient approach for the analysis and estimation of the division of rod-shaped organisms based on CRMs. We will show how CRMs allow us to reproduce observed correlations between key cell-size variables for the adder strategy, as well as time dynamics of the cell size distribution, which are unavailable for traditional DSMs.

Our splitting rate function (*h*) is assumed proportional to the current cell-size. With this *h*, we build a continuous time Markov chain (CTMC) which transient dynamics can be estimated numerically using the finite state projection (FSP)[14] approach. FSP maps the infinite set of the states $n\in \mathbb {N}$ of a Markov chain onto a set with a finite number of states (for example *n*∈{0,1,2,3,4}). The transient probability distribution of such finite state Markov chain can approximated by using standard numerical ODE solvers.

## Methods

### CRM of bacteria cell-size transient dynamics

Consider a bacterial cell growing exponentially in size (*s*(*t*)) as

1 $$ \frac{ds(t)}{dt}=\mu s(t), \quad s(0)=s_{0},  $$

where *μ* is the cell growth rate with individual cell-size doubling time *τ*= ln2/*μ*. *s*_0_ is the initial size of the cell. Let the cell divide at time *t*_1_; then the size after division (assuming no partitioning errors) is given by

2 $$ s(t)=\frac{s_{0} e^{\mu t_{1}}}{2}e^{\mu (t-t_{1})}, \quad t > t_{1}.  $$

After *n*(*t*) divisions, the size can be written as

3 $$\begin{array}{*{20}l} s(t)&=\frac{s_{0} e^{\mu t_{1} }}{2}\prod_{i=2}^{n(t)}\frac{e^{\mu (t_{i}-t_{i-1})}}{2}e^{\mu (t-t_{n})}, \quad t > t_{n} \dots >t_{1}>0, \end{array} $$

4 $$\begin{array}{*{20}l} &=\frac{s_{0} e^{\mu t}}{2^{n(t)}}. \end{array} $$

Hence, the cell size dynamics can be rewritten as the dynamics of the counting process *n*(*t*). Let the rate of the counting process *n*(*t*) be

5 $$ h(t)=ks(t),  $$

As we show in Additional file 1, using this rate, we conclude that the size at division in a cell cycle given the newborn size *s*_*b*_ is an exponential random variable with probability distribution

6 $$ \rho(s_{d}|s_{b})=\rho(\Delta)=\frac{1}{\bar{\Delta}}\exp\left(-\frac{\Delta}{\Delta_0}\right),  $$

where *Δ*=*s*_*d*_−*s*_*b*_ is the the added size, and $\overline {\Delta }=\frac {\mu }{k}$. By this result we get:

7 $$ \mathbb{E}(s_{d}|s_{b})=\mathbb{E}(\Delta)+s_{b}=\bar{\Delta}+s_{b},  $$

which corresponds to an adder DSM model with average added size $\bar {\Delta }$. Next, we present the transient dynamics of the size distribution that can be obtained using this CRM. Further details describing this CRM have been published in past studies[15].

## Results

### Cell-size transient distribution for the adder strategy

Let *P*_*i*_(*t*) represent the probability of the counting process *n*(*t*) being in the state *n*(*t*)=*i* (cell divided *i* times at time *t*) and the transition rate *h*=*ks* with *s* given by (3). Then, the master equation that describes the dynamics of *P*_*i*_(*t*) is given by

8 $$\begin{array}{*{20}l} \frac{dP_{0}(t)}{dt}&=-ksP_{0}(t)=-ks_{0}e^{\mu t}P_{0}(t),\\ \frac{dP_{i}(t)}{dt}&=\frac{ks_{0}e^{\mu t}}{2^{i-1}}P_{i-1}(t)-\frac{ks_{0}e^{\mu t}}{2^{i}}P_{i}(t), \quad P_{i}(0)=\delta_{i,0}, \end{array} $$

where *δ*_*i*,*j*_ is the Kronecker delta. The solution for *P*_*i*_(*t*) knowing *P*_*i*−1_(*t*) is given by

9 $$\begin{array}{*{20}l} P_{i}(t)&=\frac{ks_{0}}{2^{(i-1)}}\exp\left[-\frac{ks_{0}}{\mu 2^{i}}e^{\mu t}\right]\int_{0}^{t} K(t')P_{i-1}(t')dt', \end{array} $$

where

10 $$\begin{array}{*{20}l} K(\tau)\,=\,\exp\left[\mu\tau\,+\,\frac{ks_{0}}{\mu 2^{i}}e^{\mu\tau}\right], \!\!\!\quad P_{0}(t)\,=\,\exp\left[\,-\,\frac{ks_{0}}{\mu}\left(e^{\mu t}\,-\,1\right)\right]. \end{array} $$

Analytic expressions for the first five *P*_*i*_(*t*) are shown in Additional file 1, this distribution $\vec {P}$ can be efficiently obtained, either analytic or numerical, through the solution of the truncated set of ODEs defined in (8). An numeric solution in addition to (9) can be obtained using finite state projection[14] and computing the matrix exponential associated to the master equation(8). This approach is shown in Additional file 1.

**Fig. 1:**
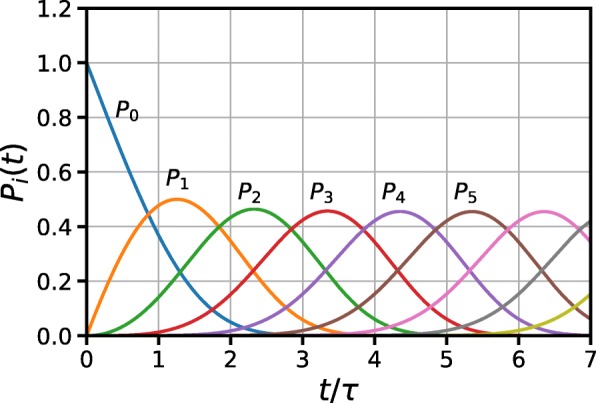
Time dynamics of the first five *P*_*i*_s defined by (9)

Once solved (9), we obtained time trends for some *P*_*i*_(*t*) which are plotted in Fig. 1.

**Fig. 2:**
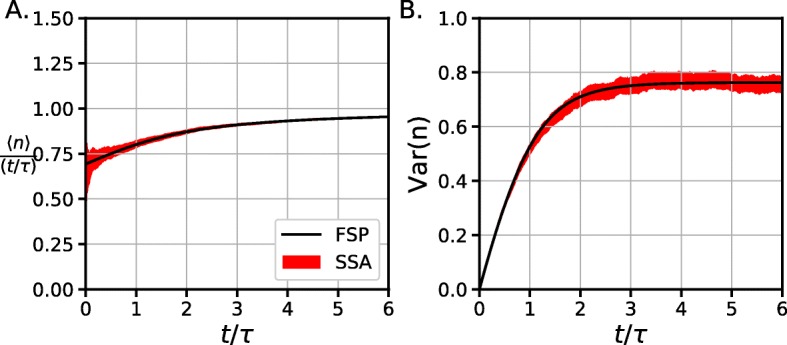
Transient dynamics of the first moments of *P*_*n*_**a**. Asymptotic behavior of 〈*n*〉 showing that ${\lim }_{t\rightarrow \infty }\langle n\rangle =\frac {t}{\tau }$. **b**. $\text {var}(n)=\sum _{n} (n-\langle n\rangle)^{2}P_{n}(t)$ reaches a steady value as *t*→*∞*. The shaded area corresponds to a 95% confidence interval of the mean and variance of 10K SSA trajectories

Using this *P*_*i*_s, the transient dynamics of the mean number of divisions $\langle n\rangle =\sum n P_{n}(t)$ and their variance $\text {var}(n)=\sum _{n} (n-\langle n\rangle)^{2}P_{n}(t)$ can be calculated. These dynamics are in perfect agreement with the results based on stochastic simulation algorithms (SSA) as can be seen in Fig. 2. After a few divisions, the distribution $\overrightarrow {P_{i}}$ reaches a mean $\langle n\rangle \rightarrow \frac {t}{\tau }$ and the variance reaches a finite limit when *t*→*∞* around 0.75 (no exact expression was calculated).

As we show in Additional file 1, in the limit of *t*→*∞* the distribution of *P*_*i*_ satisfies

11 $$\begin{array}{*{20}l} {\lim}_{i\rightarrow\infty}\Vert P_{i}(t)-P_{i-1}(t-\tau)\Vert&=0, \end{array} $$

suggesting an asymptotic invariance under translation on, simultaneously, *n*→*n*+1 and *t*→*t*+*τ*. This invariance is also satisfied by the size $s(t)=\frac {s_{0} e^{\mu t}}{2^{n(t)}}$. This property will be used to obtain the limit cell-size distribution in the following section.

### Size distribution of independent cells

Consider a set of independent cells, all of them growing exponentially at rate *μ*. We assume that once one cell divides, we only keep one of the descendant cells, the other descendant is discarded. Hence, the size population is fixed at all times. Experimentally, this is usually obtained in microfluid-based experiments like the *mother machine*[2, 16].

For simplicity, let us assume that all cells started at *t*=0 with size *s*_0_, i.e. with initial distribution

12 $$ \rho(s|t=0)=\delta(s-s_{0}).  $$

Our goal is to compute the distribution of cell sizes over the population at time *t*>0.

Using (12) and (9), the probability distribution of cell sizes after a time (*t*) of a population of independent cells is given by

13 $$ \rho(s|t)=\sum_{i=0}^{\infty}\delta\left(s-\frac{s_{0}e^{\mu t}}{2^{n}}\right)P_{i}(t).  $$

Distribution (13) corresponds to a sum of weighted Dirac delta distributions *δ*(*x*) with positions centered on sizes (3). The mean and variance of the size are given by

14 $$\begin{array}{@{}rcl@{}} \langle s(t)\rangle &=&\sum_{i=0}^{\infty}\frac{s_{0}e^{\mu t}}{2^{i}} P_{i}(t) \end{array} $$

15 $$\begin{array}{@{}rcl@{}} \text{var}(s(t))&=&\sum_{i=0}^{\infty}\left(\frac{s_{0}e^{\mu t}}{2^{i}}-\langle s(t)\rangle\right)^{2} P_{i}(t) \end{array} $$

**Fig. 3:**
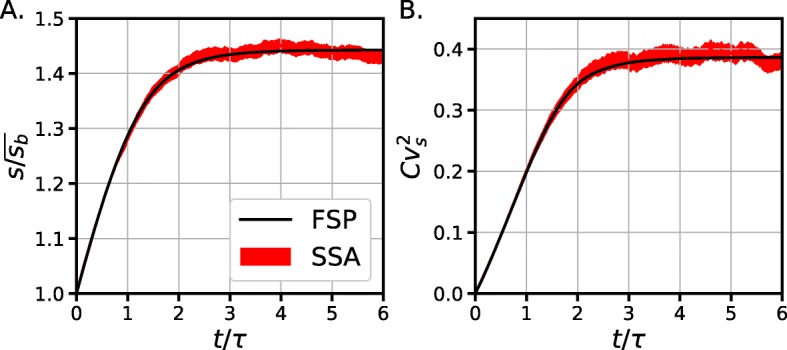
Time dynamics of size distribution *ρ*(*s*,*t*) defined by Eq. (13) with initial conditions *ρ*(*s*,*t*)=*δ*(*s*−*s*_0_). Red is the 95% confidence interval for a MonteCarlo simulation for 10000 cells (Stochastic Simulation Algorithm) and Black is the expected value obtained by the integration of *P*_*n*_(*t*) using a Finite State Projection algorithm. **a**. Expected relative mean size vs. time. **b**. Variance of size population vs. time

Figure 3 shows moment dynamics (14) projected over the ten first states (*P*_*i*_) on the time interval (0,7*τ*). Theoretical and SSA simulations over 10K cells are compared.

**Fig. 4:**
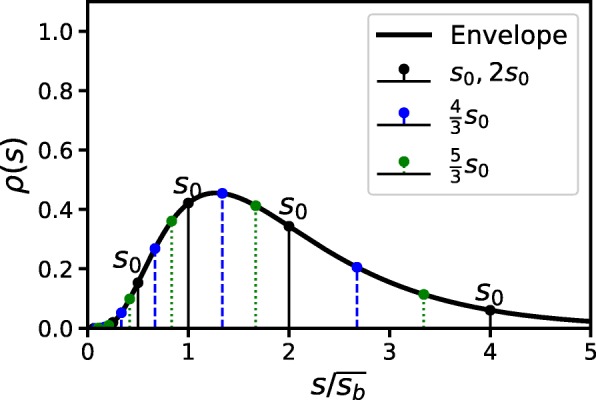
Limit *ρ*(*s*) defined as the envelope of the Dirac delta distributions for different initial conditions ($s_{0},\frac {4}{3}s_{0},\frac {5}{3}s_{0}$) after a time *t*=7*τ*. Every stem is the result of 10K SSA simulations

As consequence of the periodic conditions (11), the size distribution (13) is the same after a division time *τ*. Equivalently, for a fixed *t*, the position of the Deltas will change depending on the initial size *s*_0_. Figure 4 shows how this effect arises. Note how the deltas draw an enveloping curve changing *s*_0_ or equivalently advancing on time. Deltas of cells starting from different starting sizes (from *s*_0_ to 2*s*_0_) measured at time *t*=7*τ* are shown. These deltas are compared with data computed using SSA showing excellent agreement.

This envelope distribution could be important in future estimations of cell distributions in actual experiments.

## Discussion

Some details here are worth being discussed. First, as was pointed out previously[17], the proposed splitting rate function reproduces the *adder* DSM, this is, the observed decorrelation between the added size (*Δ*=*s*_*d*_−*s*_*b*_) and the size at birth. This behavior was found by most experimental studies[2, 16]. However, the noise in added size taken as the $CV_{\Delta }^{2}$ seems to be higher than the one experimentally observed (while our typical $CV^{2}_{\Delta }$ is 1, experimentally it is as small as 0.1). This low noise can be reached considering a multi-step process as suggested by [17], although this would make our model more complex. We will elaborate on this idea in upcoming studies.

The idea behind this control mechanism relies on the definition of a splitting rate function dependent of the size. As pointed out by some authors [2, 13], the splitting could correspond to the formation of the FtsZ ring. Here, our assumption would be that the formation of this ring has a rate proportional to the size of the bacteria. The dependence on size has been suggested by previous observations [18, 19].

Although the assumption that all cells start at a fixed size seems quite unrealistic, extensions to cases where the initial cell size correspond to a distribution can be easily done. Note that such distribution should be convoluted with the distribution obtained using our proposed approach. Some effects of a starting size distribution with finite variance are shown in additional file 1.

Extrapolation of this approach to division strategies away from the adder strategy is not too difficult. As we have shown in [15], we can get other strategies by considering a SRF that is non-linearly dependent on the size; i.e. *h*=*ks*^*λ*^. Further discussion is implemented in Additional file 1 and the full description of this approach will be done in upcoming publications.

Biological implications of this approach are extensive. Transient dynamics of cell size might unveil details on the mechanisms behind gene product homeostasis [8, 20]. Additionally, this dynamics might provide tools for quantifying the noise transmitted by the stochasticity of division events. The relationship between SRF functions and cell size control strategies further enable the use of recently proposed frameworks for gene expression[21] and cell lineage[22] analysis of experimental data from proliferating cell populations.

## Conclusions

Continuous rate models (CRM) for division control of rod-shaped bacteria are uncommon due to scarce mappings to experimental results. Here, starting from a splitting rate function proportional to the size, we explore its implication on the division control. We compute the expected number of divisions during a given time interval and its variance, and the dynamics of the size distribution of a population of independent cells.

Size dynamics of rod-shaped organisms can be described by a continous-time Markov chain. This model describes the division as a single-step process with occurrence rate proportional to the cell size. In past studies, we showed how this rate yields to an *adder* strategy which is, usually, taken as the main paradigm of cell division. Here, we explore the transient dynamics of cell size distribution considering this division strategy. Numeric estimations were done using the finite state projection algorithm.

We consider cells starting at same conditions and see how size statistics evolves. We perform some preliminary predictions like the distribution of division times and the size distribution along the time showing the evolution of mean size and its variance. We also observe that these distributions have periodic properties with an associated period of one division time.

## Supplementary information

**Additional file 1** Supplementary information.

## Data Availability

Not applicable.

## Abbreviations

CRM: Continuous rate model
CTMC: Continuous-time Markov chain
DSM: Discrete stochastic model
FSP: Finite state projection
SRF: Splitting-rate function
