# Supplementary material for: Efficient computation of stochastic cell-size transient dynamics
Source: BMC Bioinformatics. 2019 Dec 27;20(Suppl 23):647. doi: 10.1186/s12859-019-3213-7 (PMC6933677; doi:10.1186/s12859-019-3213-7)
Supplement: Supplementary file 1 — Additional file 1 Supplementary information. [file 12859_2019_3213_MOESM1_ESM.pdf]

Supplemental Information: Efficient computation of stochastic cell-size transient dynamics

The SRF integration

Let us assume that cells grow with exponential rate μ and the time dynamics for a single cell size (s) be:

$$\dot{s} = \mu s \quad s(0) = s_b \quad \dot{\tau} = 1 \tag{S1}$$

We define the timer variable τ which is reset to $\tau = 0$ every time the cell divides. Cells will continue growing until division at age τ_d.

As in [?], we define the splitting rate function (SRF) $h(s, s_b)$ defined as the local Poisson rate of cell division at time (τ_d), i.e.

$$P(\tau_d \in (\tau, \tau + d\tau)) = h(s(\tau), s_b)d\tau \tag{S2}$$

is the probability that a cell with size s and newborn size s_b divides within the time interval τ, $\tau + d\tau$.

Suppose we want to compute the probability that a cell divides while is growing from size s to $s + ds$ given the size at birth s_b. Thus, we can start from the probability that had not divided during the time interval $(0, \tau)$ and then divided during the time interval $(\tau, \tau + d\tau)$ this is at size $s(\tau) = s_d$. given the SRF (S2), we have:

$$\rho(s_d|s_b)ds = \exp\left(-\int_0^\tau h(s(\tau'))d\tau'\right) h(s(\tau))d\tau \tag{S3}$$

Most of the adder models only consider this SRF as dependent only on the added size, this is: $h(s(\tau), s_b) = h(s(\tau) - s_b) = h(\Delta(\tau))$[?, ?, ?]. Here, we will show how an adder mechanism can rise assuming a SRF dependent on the current size following the work done in previous research[?].

If we use:

$$h(s) \exp\left[-\int_0^\tau h(s(\tau'))d\tau'\right] = -\frac{d}{d\tau} \exp\left[-\int_0^\tau h(s(\tau'))d\tau'\right] \tag{S4}$$

we can rewrite the equation (S3) as:

$$\rho(s_d = s|s_b)\frac{ds}{d\tau} = -\frac{d}{d\tau} \exp\left[-\int_0^\tau h(s(\tau'))d\tau'\right] \tag{S5}$$

which, after integration, can be written as:

$$\int_0^\tau \rho(\tau_d = \tau'|s_b)d\tau' - 1 = -\exp\left[-\int_0^{\tau(s_d,s_b)} h(s(\tau'))d\tau'\right] \tag{S6}$$

After some algebra, it can be obtained:

$$\begin{aligned} h(s(\tau)) &= -\frac{d}{d\tau} \ln\left(1 - \int_0^\tau \rho(\tau_d = \tau'|s_b)d\tau'\right) \\ &= -\frac{d}{d\tau} \left(\ln(1 - P(\tau_d = \tau|s_b))\right) \end{aligned} \tag{S7}$$

Where $\rho(\tau_d = \tau|s_b)d\tau$ is the probability of, giving a size at birth s_b and the growth rate μ, cell splits during the time interval $(\tau, \tau + d\tau)$ and $P(\tau_d = \tau|s(0) = s_b)$, or simply $P(\tau_d|s_b)$, is the cumulative distribution function (CDF) for this division probability. By the relationship (S7), given the SRF, the cumulative distribution can be estimated as we can see as follows.

Simple model for Adder strategy

SRF considered here is that proportional to the current cell size (s).

$$h(s(\tau)) = ks = ks_b \exp(\mu\tau) \tag{S8}$$

With k some constant. Taking into account the exponential growth law (S1), there is a relationship between this size (s) and the cell age(τ):

$$\tau = \frac{1}{\mu}\ln\left(\frac{s}{s_b}\right) \tag{S9}$$

By the SRF given in (S8), (S7), can be solved for $\rho(\tau_d|s_b)$ as follows. We can start by solving for the CDF:

$$P(\tau_d|s_b) = 1 - \exp\left(\frac{ks_d}{\mu}(1 - e^{\mu\tau_d})\right) \tag{S10}$$

Which gives a probability density function (PDF) of cell splitting at time $\tau = \tau_d$

$$\begin{aligned}\rho(\tau = \tau_d|s_b) &= \frac{dP(\tau_d|s_b)}{d\tau} \\ &= ks_b \exp\left(\mu\tau_d + \frac{ks_b}{\mu}(1 - e^{\mu\tau_d})\right)\end{aligned} \tag{S11}$$

Using the relationship (S9), we obtain:

$$\begin{aligned}\rho(s_d|s_b) &= \rho(\tau_d|s_b)\frac{d\tau}{ds} = \frac{k}{\mu}\exp\left(-\frac{k}{\mu}(s_d - s_b)\right) \\ &= \frac{k}{\mu}\exp\left(-\frac{k}{\mu}\Delta\right)\end{aligned} \tag{S12}$$

If we define the added size $\Delta = s_d - s_b$, the distribution of this added size is given by:

$$\begin{aligned}\rho(s = s_d|s_b) &= \rho(s_d - s_b) = \rho(\Delta) \\ &= \frac{k}{\mu}\exp\left(-\frac{k}{\mu}\Delta\right)\end{aligned} \tag{S13}$$

This means that, by this model, the added size does not depend neither the splitting time nor the newborn size and is an exponential distributed variable with mean

$$E[\Delta] = \frac{\mu}{k} \tag{S14}$$

Exponential Matrix formalism for FSP

Starting with the matricidal notation of the master equation:

$$\frac{d\vec{P}}{dt} = \mathbf{A}\vec{P} \tag{S15}$$

Whit $\mathbf{A}$ the matrix associated to the desired sate projection. For instance, suppose that the projection is made over the first five states, then, the matrix $\mathbf{A}$ assumes the explicit form:

$$\mathbf{A} = \begin{bmatrix} -s_0 e^{\mu t} & 0 & 0 & 0 & 0 \\ s_0 e^{\mu t} & -s_0\frac{e^{\mu t}}{2} & 0 & 0 & 0 \\ 0 & s_0\frac{e^{\mu t}}{2} & -s_0\frac{e^{\mu t}}{4} & 0 & 0 \\ 0 & 0 & s_0\frac{e^{\mu t}}{4} & -s_0\frac{e^{\mu t}}{8} & 0 \\ 0 & 0 & 0 & s_0\frac{e^{\mu t}}{8} & -s_0\frac{e^{\mu t}}{16} \end{bmatrix} \quad \vec{P} = \begin{bmatrix} P_0 \\ P_1 \\ P_2 \\ P_3 \\ P_4 \end{bmatrix} \tag{S16}$$

First, integrating $\mathbf{A}$, ie, integrating element by element on the time, we verify that the commutator satisfies

$$\left[\int \mathbf{A}dt, \mathbf{A}\right] = 0 \tag{S17}$$

Because this is the case, the solution of $\vec{P}$ is a linear combination of autovectors of the matrix exponential of $\int \mathbf{A}dt$. This exponential matrix is defined as:

$$e^{\int \mathbf{A}dt} = \sum_{k=0}^{\infty} \frac{1}{k!}\left(\int \mathbf{A}dt\right)^k \tag{S18}$$

Then, if $\{\vec{a_i}\}$ are the eigenvectors of $e^{\int \mathbf{A}dt}$ and $\{\lambda_i\}$ their respective eigenvalues, $\vec{P}$ is given by:

$$\vec{P(t)} = \sum c_i \vec{a_i} \lambda_i(t) \tag{S19}$$

where the constants $\{c_i\}$ are set by the initial conditions $P_i = \delta_{i,0}$. Finally, the expressions of the 5 first P_ns are:

$$P_0(t) = \exp(-F(t)) \tag{S20}$$

$$P_1(t) = -2\exp(-F(t)) + 2\exp\left(-\frac{F(t)}{2}\right) \tag{S21}$$

$$P_2(t) = \frac{4}{3}\exp(-F(t)) - 4\exp\left(-\frac{F(t)}{2}\right) + \frac{8}{3}\exp\left(-\frac{F(t)}{4}\right) \tag{S22}$$

$$P_3(t) = -\frac{8}{21}\exp(-F(t)) + \frac{8}{3}\exp\left(-\frac{F(t)}{2}\right) - \frac{16}{3}\exp\left(-\frac{F(t)}{4}\right) + \frac{6}{21}\exp\left(-\frac{F(t)}{8}\right) \tag{S23}$$

$$P_4(t) = \frac{16}{315}\exp(-F(t)) - \frac{6}{21}\exp\left(-\frac{F(t)}{2}\right) + \frac{32}{9}\exp\left(-\frac{F(t)}{4}\right) + \frac{128}{21}\exp\left(-\frac{F(t)}{8}\right) - \frac{1024}{315}\exp\left(-\frac{F(t)}{16}\right) \tag{S24}$$

With $F(t) = \frac{ks_0}{\mu}(e^{\mu t} - 1)$.

Tanslation invariance for the P_n's

In order to check the asymptotic invariance under translation on, simultaneously,$n \rightarrow n+1$ and $t \rightarrow t+\tau$. We check computationally the property

$$\lim_{i\rightarrow\infty} \|P_n(t) - P_{n-1}(t-\tau)\| = 0 \tag{S25}$$

where we define the distance $\|f\| \equiv \int_{-\infty}^{\infty} |f(t)|dt$. This distance between $P_n(t)$ and $P_{n-1}(t-\tau)$ for different n's is plotted in Figure S1 showing the convergence stated in (S25)

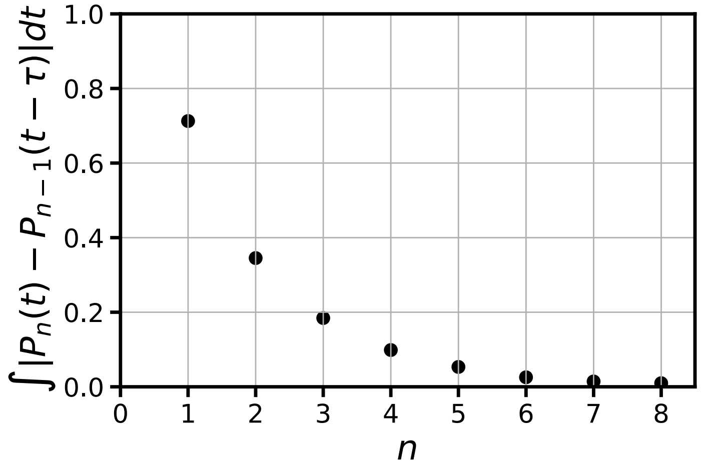

Figure S1: Convergence of $\|P_n(t) - P_{n-1}(t-\tau)\|$ to 0 as $i \rightarrow \infty$

Effects of finite variance of size starting distribution

We mentioned in the main article that it is not realistic to consider a starting condition that all the cells start at the same size. This is $\rho(s|t=0) = \delta(s - \overline{s_b})$. The most realistic scenario corresponds to a initial distribution with finite variance.

To obtain this dynamics, consider that we have solved the time dynamics for different Delta-distributed population. Let $\rho(s_b)$ be the newborn size distribution at $t = 0$, then, the size distribution along the time is given by:

$$\begin{aligned}\rho(s|t) &= \int ds_b \rho(s_b)\rho(s|t, s_b) \\ &= \int ds_b \rho(s_b) \left[\sum_{i=0}^{\infty} \delta\left(s - \frac{s_b e^{\mu t}}{2^i}\right) P_i(t, s_b)\right] \end{aligned} \quad \text{(S26)}$$

To address this condition, we simulate the size dynamics of a population of independent cells with sizes distributed by a Log-Normal distribution with chosen parameters such as $\langle s\rangle/\overline{s_b} = 1$ and $\mathrm{var}(s/\overline{s_b}) = 0.1$. The dynamics of these moments along the time is plotted in the Figure S2.

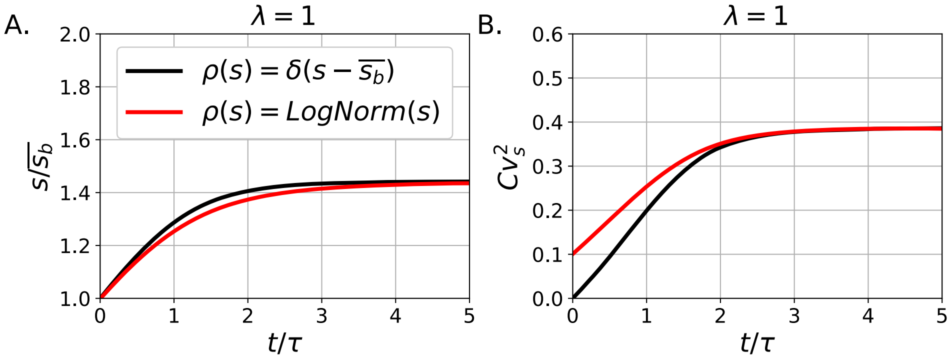

Figure S2: Effects on the size dynamics starting from a size distribution with non-zero variance. A. The mean size along the time for population of cells with different initial size distributions: (Black) Direc-Delta distribution, (Red) Log-Normal distribution B. Dynamics of the CV_s^2 along the time for different initial size distributions.

In this Figure, it can be seen how independently on the starting size distribution, these moments converges to the same stationary values with little differences in their dynamics.

Obtaining division strategies different to the adder

As we detailed in [?], to get a division strategy different from the adder, this is, either the sizer or the timer or intermediate strategies; the splitting rate function (h) could be modified including a power of the current size (s). This is:

$$h(\tau) = ks(\tau)^{\lambda} \quad \text{(S27)}$$

In figure S3, we show how changing λ lead us to obtain any division strategy. For $\lambda \to 0$ we obtain the timer, $\lambda \to \infty$ gives us the sizer and intermediate values give us intermediate division strategies.

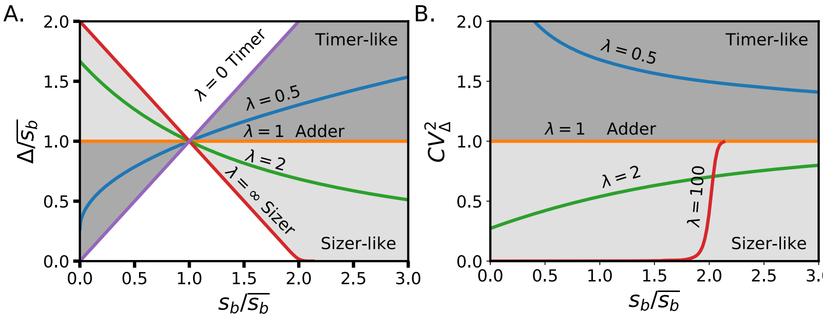

Figure S3: Obtaining other division strategies including an exponent (λ) in the SRF. A. the mapping between the added size (Δ) vs the size at birth (s_b) obtaining different strategies changing λ B. Dependence of the fluctuations over the added size as function of the size at birth for different strategies.

The application of these changes in the SRF can be implemented straightforward. These results are part of an upcoming publication.
